# A Method to Assess Target Gene Involvement in Angiogenesis *In Vitro* and *In Vivo* Using Lentiviral Vectors Expressing shRNA

**DOI:** 10.1371/journal.pone.0096036

**Published:** 2014-04-23

**Authors:** Wayne Blosser, Eliza Vakana, Lisa V. Wyss, Michelle L. Swearingen, Julie Stewart, Louis Stancato, Courtney M. Tate

**Affiliations:** 1 Oncology Research, Eli Lilly and Company, Indianapolis, Indiana, United States of America; 2 Advanced Testing Laboratories, Cincinnati, Ohio, United States of America; Bristol Heart Institute, University of Bristol, United Kingdom

## Abstract

Current methods to study angiogenesis in cancer growth and development can be difficult and costly, requiring extensive use of *in vivo* methodologies. Here, we utilized an *in vitro* adipocyte derived stem cell and endothelial colony forming cell (ADSC/ECFC) co-culture system to investigate the effect of lentiviral-driven shRNA knockdown of target genes compared to a non-targeting shRNA control on cord formation using High Content Imaging. Cord formation was significantly reduced following knockdown of the VEGF receptor VEGFR2 in VEGF-driven cord formation and the FGF receptor FGFR1 in basic FGF (bFGF)-driven cord formation. In addition, cord formation was signifcantly reduced following knockdown of the transcription factor forkhead box protein O1 (FOXO1), a protein with known positive effects on angiogenesis and blood vessel stabilization in VEGF- and bFGF-driven cord formation. Lentiviral shRNA also demonstrated utility for stable knockdown of VEGFR2 and FOXO1 in ECFCs, allowing for interrogation of protein knockdown effects on *in vivo* neoangiogenesis in a Matrigel plug assay. In addition to interrogating the effect of gene knockdown in endothelial cells, we utilized lentiviral shRNA to knockdown specificity protein 1 (SP1), a transcription factor involved in the expression of VEGF, in U-87 MG tumor cells to demonstrate the ability to analyze angiogenesis *in vitro* in a tumor-driven transwell cord formation system and in tumor angiogenesis *in vivo*. A significant reduction in tumor-driven cord formation, VEGF secretion, and *in vivo* tumor angiogenesis was observed upon SP1 knockdown. Therefore, evaluation of target gene knockdown effects in the *in vitro* co-culture cord formation assay in the ADSC/ECFC co-culture, ECFCs alone, and in tumor cells translated directly to *in vivo* results, indicating the *in vitro* method as a robust, cost-effective and efficient *in vitro* surrogate assay to investigate target gene involvement in endothelial or tumor cell function in angiogenesis.

## Introduction

Tumor angiogenesis is a complex biological process that is both costly and difficult to study, often requiring extensive use of *in vivo* methodologies. Early models of *in vitro* angiogenesis (or “cord formation”) relied on the separation of endothelial cells from cancer cells through the use of a barrier or matrix as endothelial cells were reported to undergo apoptosis when in direct contact with cancer cells [1]. Recently, advances have been made in studying angiogenesis *in vitro* through the use of various co-culture systems. Monolayer co-culture systems have since been developed where fibroblasts are added in direct contact with endothelial cells resulting in endothelial tubule formation following stimulation in response to various proangiogenic growth factors [2]. Examples of these systems include co-culturing adipose stromal cells (ASCs) with endothelial cells (ECs), human umbilical vein endothelial cells (HUVECs) with normal human dermal fibroblasts (NHDFs) and adipose derived stem cells (ADSCs) with human endothelial colony forming cells (ECFCs) [3], [4].

Of the many factors that induce angiogenesis, vascular endothelial growth factor (VEGF) and fibroblast growth factor (FGF) are two of the most potent, widely expressed and most heavily implicated in pathological angiogenesis [5]. VEGF is a diffusable, endothelial cell-specific mitogenic and proangiogenic factor that is also capable of increasing vascular permeability [6]. While one of the main functions of VEGF is to control vasculogenesis and angiogenesis in normal embryonic development, it also plays a crucial role in tumor angiogenesis as a tumor-derived paracrine angiogenesis factor [7]. The overexpression of VEGF is one of the most common drivers for tumor angiogenesis, a process that is necessary for the delivery of nutrients to and removal of waste from the tumor microenvironment. There are numerous therapeutics developed to block the activity of VEGF, through small molecule inhibition of receptor tyrosine kinase activity, antibodies that block ligand-receptor binding, and the use of soluble decoy receptors to reduce ligand binding to full length receptors [8].

FGF-2 or basic FGF (bFGF) is another important proangiogenic factor that, unlike VEGF, exerts its effects on a variety of cell types including endothelial cells, smooth muscle cells and neurons [7]. It can exert its activity as an endogenous (intracrine) or exogenous (auto-/paracrine) factor by accumulating in the cytoplasm and nucleus of neuronal cells such as adrenal medullary cells [9] or by direct binding of the various FGFR isoforms initiating intracellular signaling. For the purpose of angiogenesis, endothelial cells appear to be the key player due to their responsiveness to both VEGF and bFGF and are often the target of anti-angiogenic therapies [10]–[12]. bFGF induces endothelial cell migration, proliferation and tube formation *in vitro* and is highly angiogenic *in vivo* [7]. The proangiogenic function of bFGF in tumor development is thought to be a consequence of the organism's self-repair mechanism that is initiated when tumors invade and degrade the extracellular matrix thereby releasing sequestered bFGF [13]. Once released, bFGF induces plasminogen activator (PA) and collagenase production by endothelial cells which aids in the initiation of angiogenesis [14].

In addition to VEGF and FGF receptors, two transcription factors known to be involved in VEGF mediated angiogenesis were evaluated in this study, forkhead box protein O1 (FOXO1); which directly stimulates angiogenesis and blood vessel stabilization [15], [16], and the transcription factor specificity protein 1 (SP1), which when activated causes the overexpression of various angiogenic molecules resulting in tumors that are highly angiogenic and aggressive [17]. We utilized a previously described *in vitro* ADSC/ECFC co-culture system [18] that allows the different cell types to interact, similar to the stromal environment, whereby one cell type migrates to form tubes (ECFCs) while the other serves as a feeder layer (ADSCs) that can differentiate into pericyte-like cells, expressing the pericyte differentiation marker smooth muscle actin (SMA), that ultimately envelop the tubules. Cord formation was assessed using High Content Imaging [19]–[21] following knockdown of VEGF and bFGF receptors (VEGFR2 and FGFR1, respectively) as well as the transcription factors FOXO1 in the ADSC/ECFC co-culture system and SP1 in a tumor driven co-culture system using lentiviral delivered short hairpin RNA (shRNA). Lentiviral shRNA was used to create non-selected ADSC/ECFC stable pools for the *in vitro* co-culture assay and non-selected stable pools of U-87 MG in the *in vitro* tumor driven co-culture assay. Additionally, using lentiviral delivered shRNA allowed for stable knockdown of VEGFR2 and FOXO1 in puromycin-selected ECFCs, to investigate the effect on *in vivo* angiogenesis in a Matrigel plug assay. To demonstrate the ability to interrogate tumor angiogenesis *in vitro* and *in vivo*, U-87 MG cells with stable SP1 knockdown were puromycin selected and used for the *in vitro* tumor-driven cord assay as well as for *in vivo* tumor angiogenesis studies. The marrying of lentiviral delivered shRNA with an *in vitro* and functional *in vivo* model of angiogenesis is a powerful technique for studying the potential proangiogenic role of virtually any protein. This system is robust, reproducible and cost effective enough to screen for proangiogenic factors *in vitro*, and assessment of these same proteins in an *in vivo* model of functional angiogenesis. We therefore think our system represents a significant advance over currently available mono and co-culture angiogenesis assays.

## Results and Discussion

### shRNA knockdown of VEGFR2, FGFR1 or FOXO1 reduced growth factor-driven *in vitro* cord formation

Lentiviral delivery of shRNA has become an increasingly important tool in recent years due to its ability to transduce both dividing and non-dividing cells thereby allowing the shRNA targeting sequence to stably integrate into the genome for consistent and long-term knockdown. Here, we provide a method using lentivirus to deliver shRNA sequences against growth factor receptors and transcription factors in order to study the respective gene's function as it pertains to cord formation *in vitro* and *in vivo*.

To establish proper controls for the *in vitro* cord formation assay, we selected shRNA constructs targeting VEGFR2 for VEGF driven cord formation, FGFR1 for bFGF driven cord formation and FOXO1 for both VEGF and bFGF driven cord formation. VEGFR2 was chosen over other VEGF receptors as it is exclusively expressed in endothelial cells and plays a major role in tumor vascularization, growth and metastasis [6]. In addition, multiple studies have shown through the use of antisense oligomers and dominant-negative mutants that VEGF binding to VEGFR2 compared to VEGFR1 is a critical requirement to induce the full spectrum of VEGF driven biological responses [22]-[24]. Through various splice variants there is great diversity among the FGF receptor family but the two receptors that are indispensable in normal development are FGFR1 and FGFR2; disruption of either gene leads to early embryonic death [25]. We focused on FGFR1 due to the autocrine and paracrine signalling that occurs when binding bFGF, its high affinity ligand [26]. The transcription factor FOXO was used as an additional control due to its essential role in vascular development, where it has been observed that FOXO1 KO mice are embryonic lethal, due to the inability of emerging blood vessels to properly develop [27], [28].

**Figure 1:**
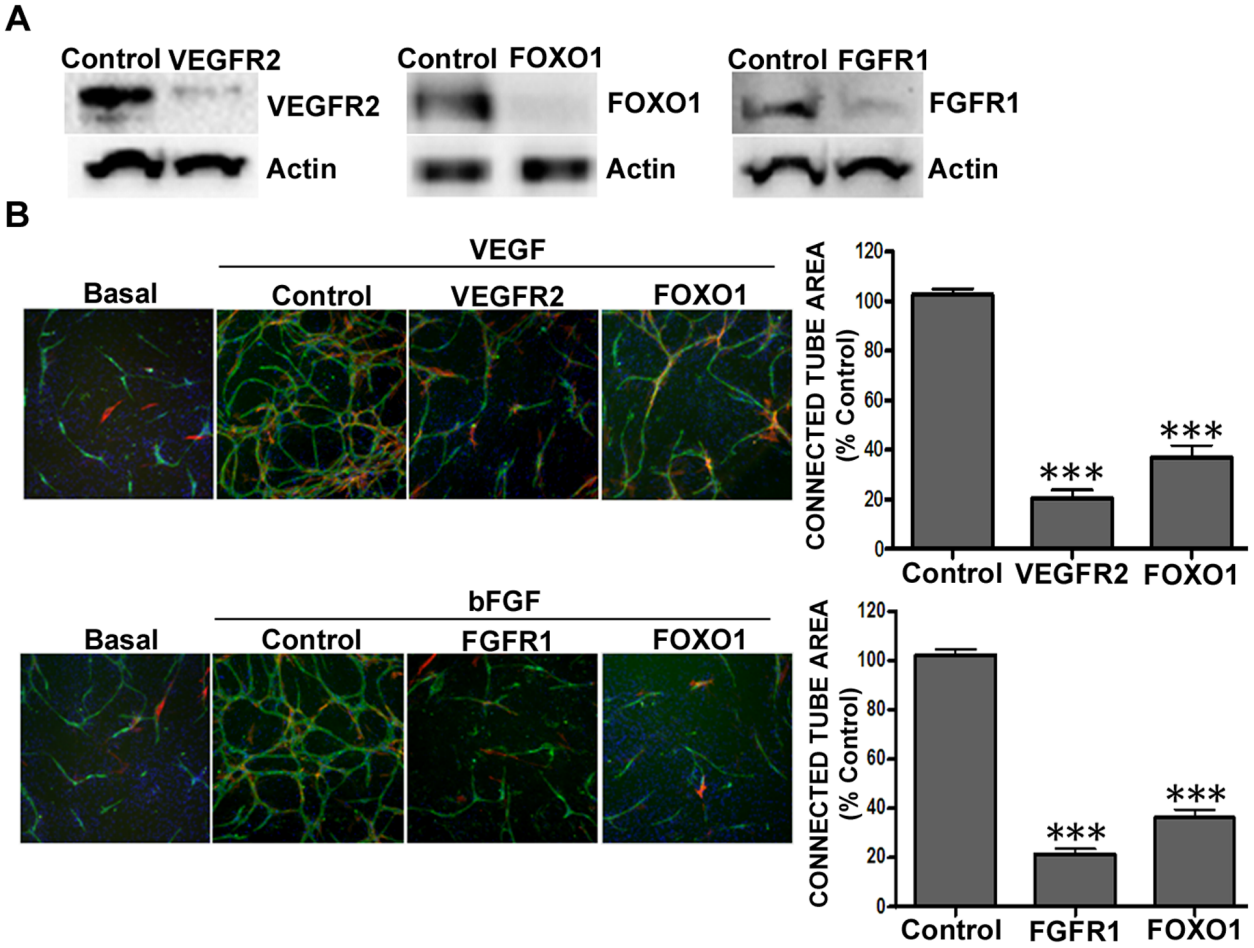
Reduction in VEGFR2, FGFR1, or FOXO1 expression in ADSCs/ECFCs reduced growth factor-driven *in vitro* cord formation. (AB); ADSCs/ECFCs were transduced with non-targeting (control), or pooled shRNA directed against VEGFR2, FOXO1, or FGFR1, for 72 hours and (A) whole cell protein extracts were isolated and subjected to Western blot analysis using antiserum against VEGFR2, FOXO1 and FGFR1 (82.1±5.9, 91.5±2.7 and 73.7±7.1% knockdown respectively), using β-actin as a loading control, or (B) analyzed for cord formation with PBS (Basal), 10 ng/ml VEGF, or 15 ng/ml bFGF stimulation for 72 hours before immunohistochemistry for CD31 (green), α-smooth muscle actin (red), and Hoechst to stain nuclei (blue). Representative images (5× magnification) are shown. Graphs represent mean ± standard error from three independent experiments, and asterisks denote statistically significant differences (*, *p*<0.05; **, p<0.01; ***, p<0.001) compared to non-targeting shRNA controls.

For analysis of the effects of shRNA knockdown on specific known proangiogenic factors, the ADSC/ECFC co-culture system was virally transduced (multiplicity of infection (MOI) of ∼9) prior to the addition of growth factors. Following knockdown with lentiviral shRNA constructs, immunoblot results showed significant knockdown of VEGFR2 (p<0.001; 82.1±5.9% protein reduction), FGFR1 (p<0.001; 91.5±2.7% protein reduction) and FOXO1 (p<0.001; 73.7±7.1% protein reduction) compared to the non-target control vector (Fig. 1A). As VEGFR2 signaling is an important component of ECFC biology, we asked whether knockdown of this receptor negatively affected cell viability. Importantly, ECFC viability following VEGFR2 knockdown, as assayed by trypan blue exclusion immediately prior to *in vivo* implantation, was largely unaffected (non-targeting control vector transduced cells at 93.4±1.2% viability; shVEGFR2 viability at 87.4±4.1%) indicating that the transduced cells were suitable for supporting cord formation *in vitro* and *in vivo* (cord formation requires actively dividing, high viability ECFCs, not ADSCs). The non-targeting control contains an shRNA insert that does not target any known human or mouse genes, making it useful as a negative control [29]. Determination of transduction efficiency via lentiviral expression of GFP was ∼83% in the ECFCs; ∼62% in the non-dividing ADSCs; and ∼75% in the U-87 MG cells as assessed using high content imaging (*data not shown*). Knockdown of VEGFR2 or FGFR1 in the presence of their respective growth factors significantly inhibited (p<0.001) *in vitro* cord formation as measured by connected tube area, which is calculated from cluster of differentiation 31 (CD31) positively stained endothelial cells (Fig. 1B). In addition, knockdown of FOXO1 yielded a significant reduction (p<0.001) in cord formation for both VEGF and bFGF driven systems (Fig.1B). It is important to note that nuclei counts were captured during image acquisition to show that knockdown of the target genes did not affect growth of the ADSC feeder layer (data not shown).

### shRNA knockdown of VEGFR2 or FOXO1 reduced *in vivo* vessel formation

**Figure 2:**
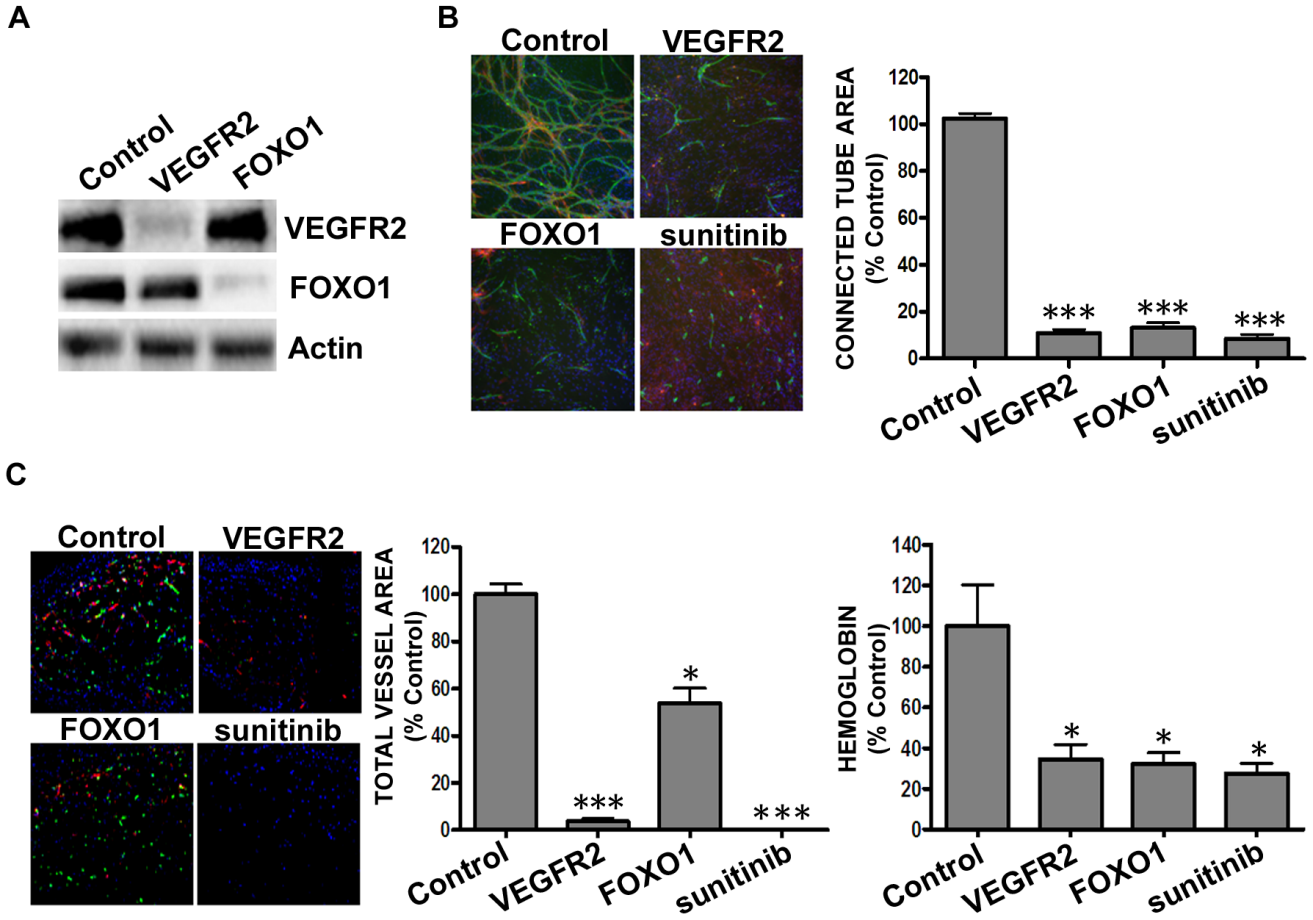
Reduction in VEGFR2 or FOXO1 expression in ECFCs reduced hemoglobin content and vascularization *in vivo*. (A-B) ECFCs were transduced with non-targeting (control), or pooled shRNA directed against VEGFR2 or FOXO1 and (A) whole cell protein extracts were isolated and subjected to Western blot analysis using antiserum against VEGFR2 and FOXO1 (85.2±7.7 and 85.1±3.9% knockdown respectively), using β-actin as a loading control, or (B) were over seeded onto ADSCs for 4 hours prior to 10 ng/ml VEGF stimulation for 72 hours before immunohistochemistry for CD31 (green), α-smooth muscle actin (red), and Hoechst dye to stain all nuclei (blue). As a control, ADSC/ECFC co-cultures were treated simultaneously with 10 ng/ml VEGF and 100 nM sunitinib. Representative images (5× magnification) are shown. Graphs represent mean ± standard error from three independent experiments, and asterisks denote statistically significant differences (*, *p*<0.05; **, p<0.01; ***, p<0.001) compared to non-targeting shRNA controls. (C-D) ECFCs transduced with non-targeting (control) or pooled shRNA directed against VEGFR2 or FOXO1 were mixed with ADSCs and co-implanted subcutaneously into the flanks of athymic nude mice. Oral dosing of a subset of mice began 4 hours prior to cell implantation and occurred twice daily with sunitinib (25 mg/kg). After 5 days of dosing, Matrigel plugs were removed and hemoglobin was quantified (C), and vasculature was visualized and quantified with immunohistochemistry (D) for human CD31 (green), anti-SMA (myofibroblasts, red), and Hoechst to stain nuclei (blue). Graphs indicate mean ± standard error from one experiment (n = 8), and asterisks denote statistically significant differences (*, *p*<0.05; **, p<0.01; ***, p<0.001) compared to non-targeting shRNA control vector.

While VEGF is not the only factor responsible for tumor angiogenesis, it is a primary driver of neovascularization for solid tumor growth and development [30]. *In vitro* cord formation is a transient assay that used stably transduced non-puromycin selected pooled cell populations of ECFCs. However, to determine the effects on vascularization *in vivo*, ECFCs were puromycin selected to produce an essentially homogenous cell population containing shRNAs targeting VEGFR2 or FOXO1. The stably selected ECFC cell populations showed significant reduction of both VEGFR2 (p<0.001; 85.2±7.7% protein knockdown) and FOXO1 (p<0.001; 85.1±3.9% protein knockdown) (Fig. 2A). Sunitinib, a multi-targeted receptor tyrosine kinase small molecule inhibitor with an anti-angiogenic mechanism of action, served as a positive control for the assay [31]–[33]. There was a significant reduction (p<0.001) in vascularization when VEGFR2 or FOXO1 was stably knocked down in ECFCs (Fig. 2B), mirroring what was previously observed with non-selected ADSC/ECFCs cell populations *in vitro* (Fig. 1), and indicating that stable gene knockdown in endothelial cells alone was feasible. Effective stable gene knockdown was critical as it demonstrated that the observed shRNA effects were specifically working through endothelial cell biology and not via effects on the ADSC feeder layer.

Implantation of the ADSC/ECFCs in an *in vivo* Matrigel plug assay was previously developed to analyze compound effects on neovascularization which in this system is primarily driven by VEGF [19], [20]. Stably transduced ECFCs were mixed with ADSCs in growth factor reduced Matrigel and implanted into nude mice to determine the effects of shRNA gene knockdown on neovascularization. Matrigel plugs were removed and analyzed for structural and functional angiogenesis through CD31 vessel staining and hemoglobin content, a measure of functional vasculature, respectively. VEGFR2 or FOXO1 knockdown significantly reduced vessel area (p<0.001 and p<0.05 respectively) and hemoglobin content (p<0.05, Fig. 2C) *in vivo* compared to non-targeting shRNA vector and comparable to the effects following sunitinib treatment (Fig. 2C). This confirms previous results that the ADSC/ECFC Matrigel plug assay is primarily a VEGF driven model of angiogenesis and therefore confirms the utility of our method to analyze gene knockdown effects on *in vivo* neoangiogenesis.

### shRNA knockdown of SP1 in U-87 MG cells reduced tumor-driven *in vitro* cord formation and *in vivo* tumor angiogenesis

**Figure 3:**
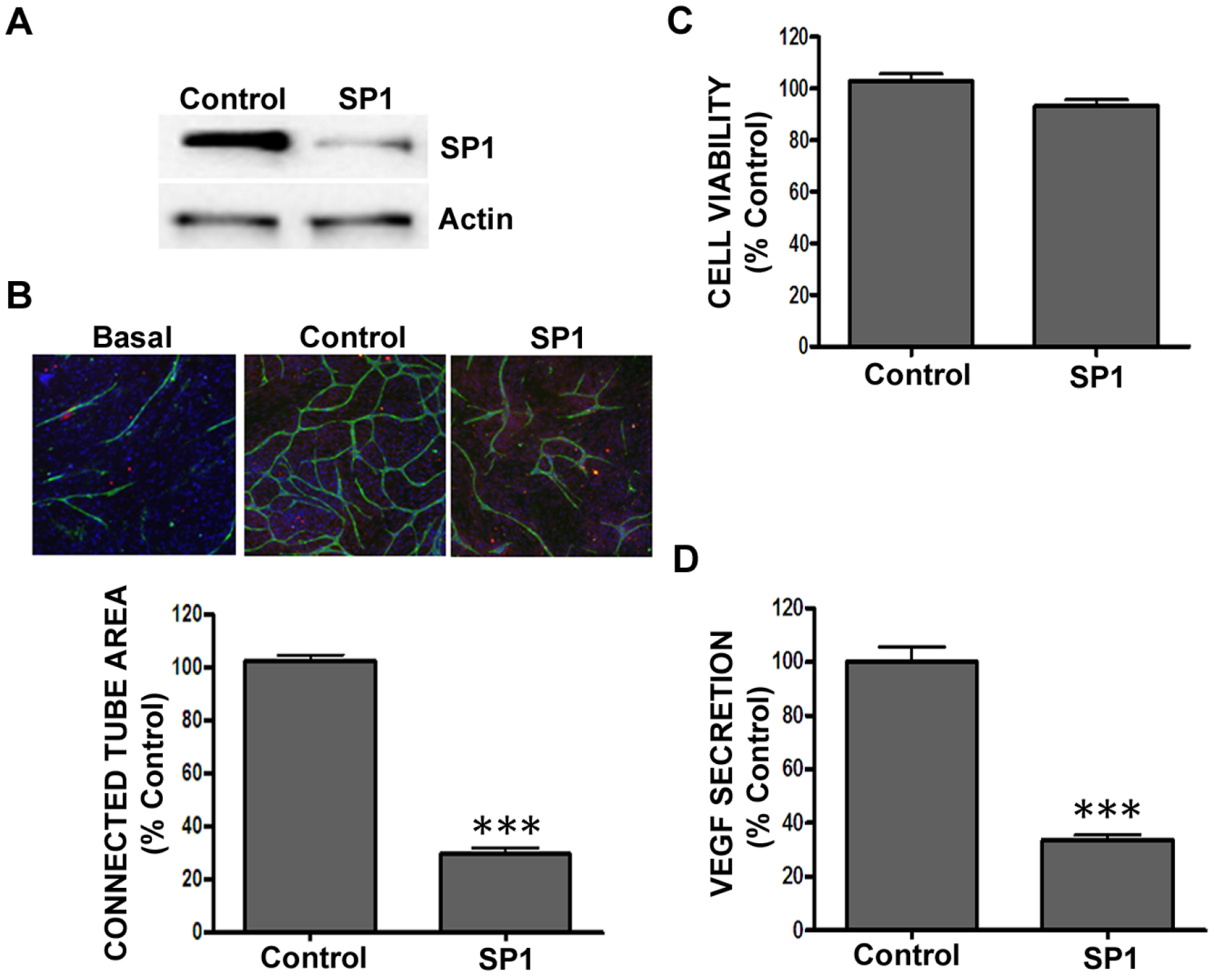
Reduction in SP1 expression in U-87 MG cells (in cord assay) reduced tumor-driven cord formation. (A-B) U-87 MG cells were plated in permeable transwells and transduced with non-targeting shRNA (control) or pooled shRNA directed against SP1 for 72 hours prior to (A) whole cell protein extract isolation and Western blot analysis using antiserum directed against SP1 (61.3±9.0% knockdown) and β-actin as a loading control, and (B) movement of the U-87 MG cells into ADSC/ECFC co-culture receiver plates. Cord formation was assessed following 72 hours by immunohistochemistry for CD31 (green), α-smooth muscle actin (red), and Hoechst to stain nuclei (blue). Representative images (5× magnification) are shown. (C) U-87 MG cell viability assesment by cellular ATP production using Cell Titer Glo Assay. (D) ELISA analysis for VEGF secretion in U-87 MG conditioned media. Graphs represent mean ± standard error from three independent experiments, and asterisks denote statistically significant differences (*, *p*<0.05; **, p<0.01; ***, p<0.001) compared to the non-targeting shRNA control vector.

In addition to analyzing the effect on angiogenesis following gene knockdown in endothelial cells, we developed an *in vitro* and *in vivo* lentiviral shRNA system to analyze angiogenesis effects following gene knockdown in tumor cells. The purpose was to more closely represent what occurs *in vivo* whereby tumors secrete soluble factors that affect the local growth environment and reduce blood vessel formation. As opposed to the aformentioned co-culture assay, whereby exogenous growth factors (VEGF, bFGF and/or EGF) are added to the assay to induce cord formation, the tumor-driven cord formation assay utilizes the factors secreted from the tumor cells in order to drive the formation of cords and is closer to the pathological angiogenesis observed in growing human tumors. In this experimental set-up, the filter between the upper chamber (tumor cells) and lower receiver chamber allows secreted proteins from the tumor cells to diffuse and induce cord formation of the ADSC/ECFC co-culture in the lower chamber. In this fashion, we are able to knockdown genes in the tumor cells and observe changes in the ADSC/ECFC co-culture as they pertain to cord formation. As VEGF expression is highly upregulated in glioblastoma (GBM) [34], we used the GBM cell line U-87 MG to assess tumor driven angiogenesis following lentiviral shRNA knockdown of SP1, a key transcription factor involved in the expression of VEGF [17], [35] that has been implicated in altered angiogenic phenotypes across multiple tumor histologies including gastric, non-small cell lung and pancreatic [36]–[38]. U-87 MG cells secrete significant levels of VEGF and are therefore capable of supporting cord formation [18]. We chose to examine VEGF secretion from the U-87 MG tumor cells as an additional measure of functional SP1 gene knockdown [17], [35]. Immunoblotting results showed significant knockdown of SP1 protein (p<0.01; 61.3±9.0% protein reduction) in U-87 MG cells transduced with SP1 shRNA, which led to a significant reduction (p<0.001) in cord formation compared to non-targeting control shRNA (Fig. 3A-B). Subsequently, VEGF secretion from U-87 MG cells was measured via colorimetric ELISA (Fig. 3D) and was significantly reduced (p<0.001) in SP1 shRNA transduced cells, thus confirming prior reports of SP1's involvement in VEGF secretion [17]. Importantly, the cord reduction observed with SP1 knockdown was not due to a reduction in tumor cell number compared to the non-targeting control (Fig. 3C).

**Figure 4:**
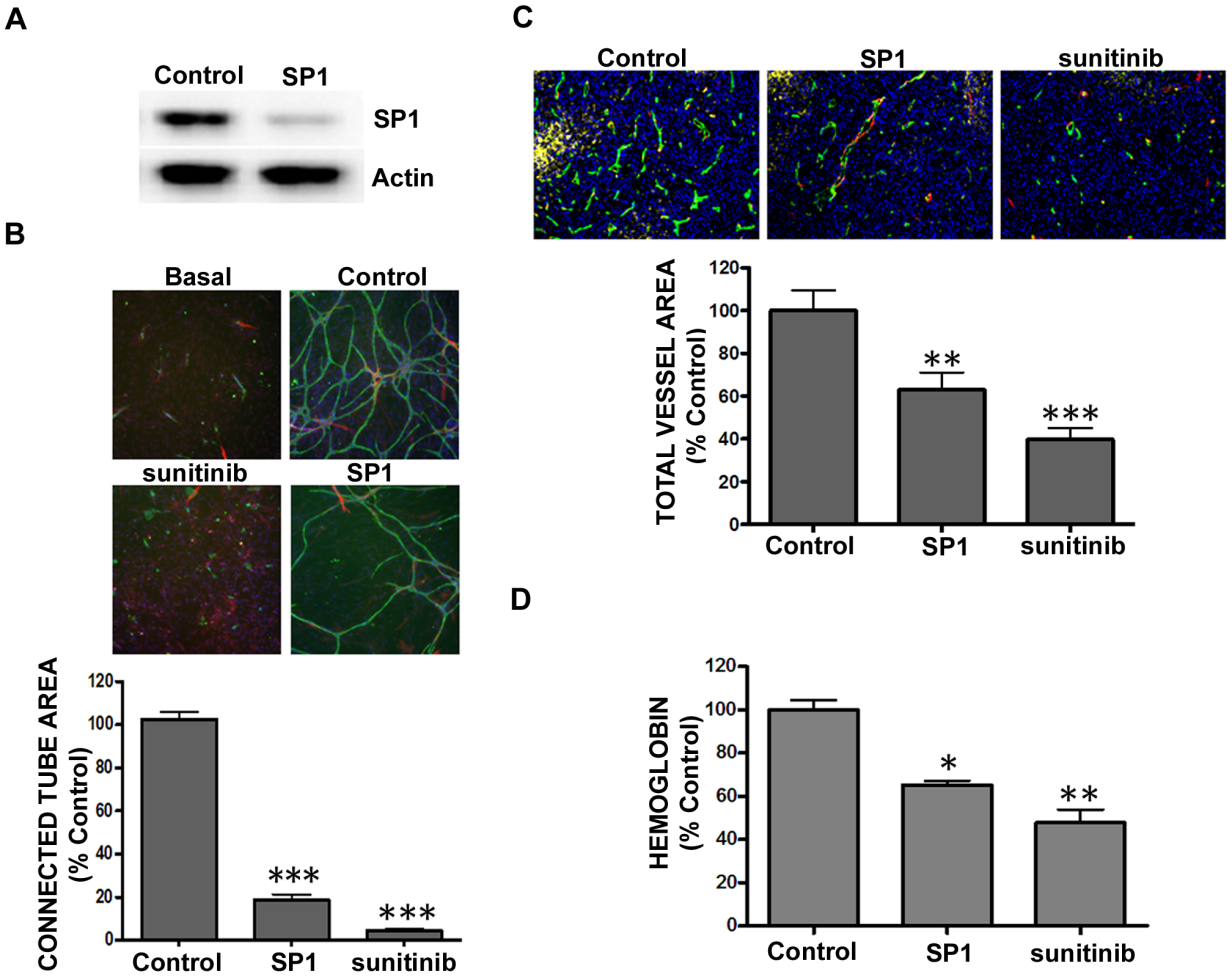
Reduction in SP1 expression in U-87 MG cells reduced tumor angiogenesis. (A-B) U-87 MG cells were transduced with non-targeting shRNA (control) or pooled shRNA directed against SP1 prior to (A) whole cell protein extract isolation and Western blot analysis using antiserum directed against SP1 (79.5±3.7% knockdown) and β-actin as a loading control, and (B) plating into permeable transwell plates above ADSC/ECFC co-culture receiver plates. Cord formation was assessed following 72 hours by immunohistochemistry for CD31 (green), α-smooth muscle actin (red), and Hoechst dye to stain all nuclei (blue). Representative images (5× magnification) are shown. Graphs represent mean ± standard error from three independent experiments, and asterisks denote statistically significant (*, *p*<0.05; **, p<0.01; ***, p<0.001) differences compared to the non-targeting shRNA control vector. (C) U-87 MG cells transduced with non-targeting shRNA (control) or shRNA directed against SP1 were implanted subcutaneously into the flanks of athymic nude mice. Oral dosing of mice with sunitinib (40 mg/kg) began when tumors reached ∼300 mm^3^ and occurred once daily. After 6 days of dosing, tumors were removed and (C) hemoglobin was quantified and (D) vasculature was visualized by immunofluorescence for CD31 (green), α-smooth muscle actin (red), GLUT1 (yellow), or Hoechst to stain all nuclei (blue). Quantitative tissue imaging was done by automated microscopy to assess tumor vascularization. Graphs (C-D) indicate mean ± standard error from one experiment (control, n = 14; SP1, n = 16; sunitinib, n = 9), and asterisks denote statistically significant differences (*, *p*<0.05; **, p<0.01; ***, p<0.001) compared to non-targeting shRNA control vector.

In addition to the non-selected U-87 MG cell pool used for the *in vitro* knockdown experiments (Fig. 3), puromycin selected stable pools of U-87 MG cells were generated with shRNA against a non-targeting control or SP1 and analyzed for *in vitro* cord formation along with implantation into nude mice to analyze tumor-driven angiogenesis. SP1 protein was shown to be significantly reduced (p<0.01; 79.5±3.7% protein knockdown) compared to the non-targeting control when assessed by Western blot (Fig. 4A). As with the data in Fig. 3, *in vitro* cord formation was significantly reduced (p<0.001) in U-87 MG cells following stable knockdown of SP1 or upon sunitinib treatment compared to non-targeting control (Fig. 4B). The puromycin selected tumor cells were then used for *in vivo* tumor angiogenesis studies in a Matrigel plug assay described previously [19], [20]. The U-87 MG tumors displaying stable SP1 knockdown had significantly reduced hemoglobin content (p<0.05) and total vessel area (p<0.01) compared to non-targeting control tumors (Fig. 4C and D). Results from the *in vivo* and *in vitro* experiments support each other with respect to reduction in vessel area/cord formation following SP1 knockdown providing further support for using the *in vitro* assay as a surrogate to the *in vivo* assay. Most importantly, SP1 knockdown ultimately resulted in reduced hemoglobin content *in vivo*.

**Figure 5:**
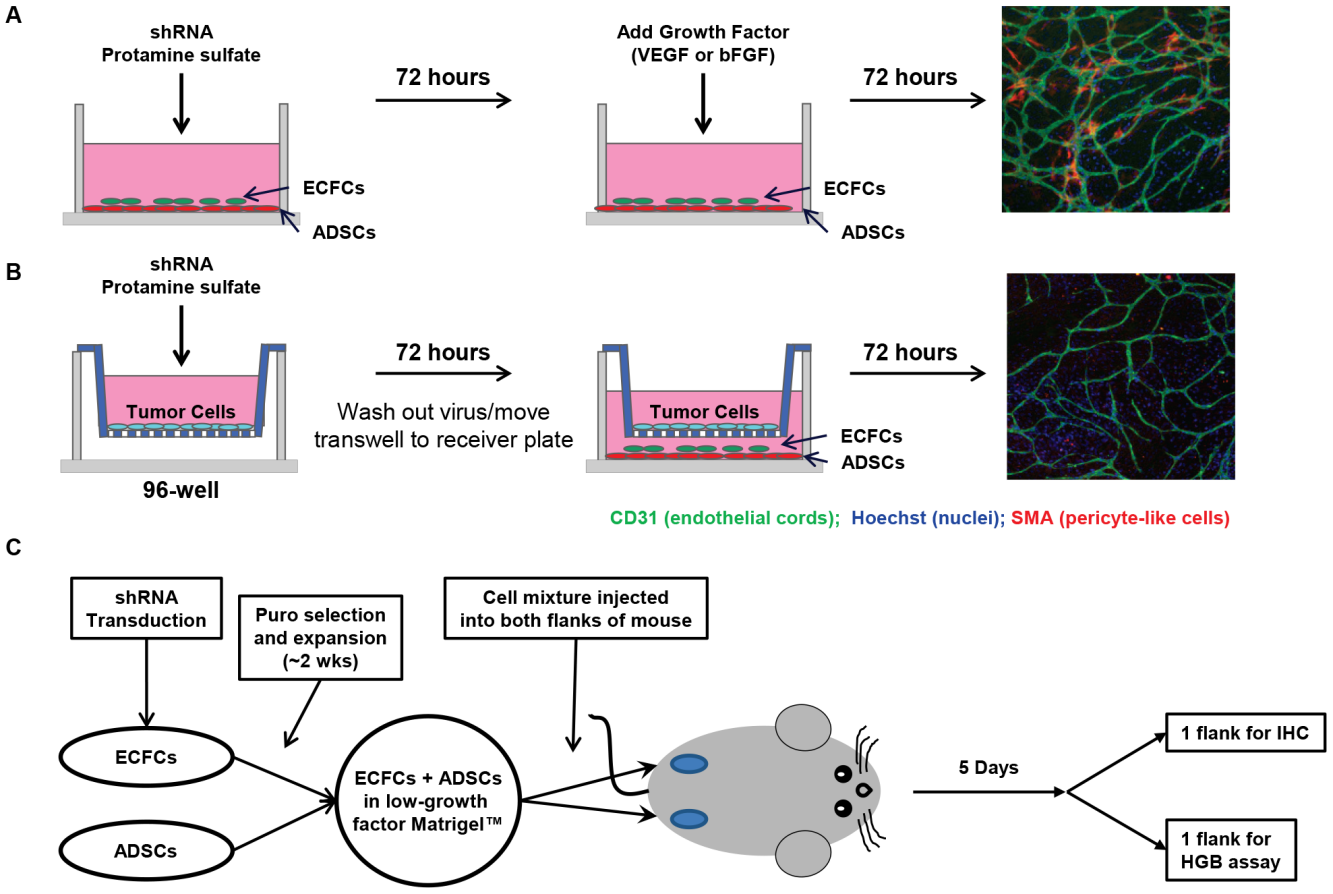
Schematic overview of the *in vitro* and *in vivo* assays. (A-B) *In vitro* cord formation assays, (A) in growth factor driven cord formation ADSCs and ECFCs are grown in co-culture and co-transduced with shRNA targeting a specific gene or a non-targeting control. Stimulation of cord formation occurs with the addition of VEGF or bFGF (other growth factors have also been successfully tested- see details in the text; (B) in tumor driven cord formation tumor cells are plated separately in a transwell plate and transduced with shRNA targeting a specific gene or non-targeting control. Following 72 hours of incubation, virus is washed out and the transwell is moved into the ADSC/ECFC co-culture receiver plate where the tumor cells sit above, but physically separate from the ADSC/ECFC co-culture. Cord formation is driven by soluble factors secreted by the tumor cells; (C) For the *in vivo* assay, ECFCs are transduced with shRNA targeting a specific gene or non-targeting control. A stable expression cell pool is generated through antibiotic selection with puromycin. Stably transduced ECFCs are then mixed with ADSCs in low-growth factor Matrigel and implanted into both flanks of a nude mouse. After 5 days the angiogenic plugs are removed with one plug slated for IHC analysis and the other plug analysed for hemoglobin content.

In summary, we have developed a method to evaluate the role of transcription factors and other protein families in tumor angiogenesis by providing a robust *in vitro* surrogate assay that mirrors *in vivo* cord formation and therefore supports the use of the *in vitro* method to interrogate transcription factors involved in angiogenesis (see Fig. 5 for a schematic). This *in vitro* cord formation method is amenable to medium-throughput analysis which allows us to screen for novel transcription factors and other protein families potentially involved in angiogenesis in a much less time intensive, cost effective way as compared to running *in vivo* angiogenesis assays. Furthermore this system has been validated for the use of VEGF, EGF, FGF, HGF, PDGF and IL6 in establishing cords *in vitro*, and therefore represents a useful system for assessing protein knockdown on angiogenesis for a wide range of growth factors/cytokines. In addition to the *in vitro* assay, this is the first time knockdown *in vivo* has been reported for the ADSC/ECFC Matrigel plug assay, which serves as a “bridge” experiment between the *in vitro* cord formation assay and analysis of neovascularization *in vivo*. Importantly, our results provide evidence to the validity of our *in vitro* co-culture cord formation assay as it translates directly to *in vivo* results. The expression of SMA in this assay further supports the notion that this co-culture system is more “physiologically relevant” than other in vitro angiogenesis assays that do not use a differentiable supporting cell type (such as the ADSC feeder layer), especially as we also see SMA-positive cells *in vivo* (which are highly likely to represent true pericytes given their co-association with both tumor and Matrigel plug CD31 staining structures). Additionally, we have provided a method whereby transcription factor (and virtually any gene family) involvement in angiogenesis can be evaluated through the use of lentiviral shRNA to knockdown desired genes of interest. Our findings support the use of this *in vitro* method to interrogate gene target knockdown in the cord formation assay to determine genes of interest involved in angiogenesis. On-going work in this system includes screening and validating the knockdown of over 400 additional transcription factors with known and unknown angiogenic function. Using these techniques we have identified several transcription factors not previously reported to be involved in angiogenesis, results of which will be published in a future manuscript.

## Materials and Methods

### Cell culture

U-87 MG cells (American Type Culture Collection (ATCC), Manassas, VA) were grown according to ATCC guidelines in Eagle's Minimal Essential Media (Hyclone Laboratories, Logan, UT) supplemented with 10% FBS (Invitrogen, Carlsbad, CA). Adipose derived stem cells (ADSCs, Zen-Bio, Research Triangle Park, NC) and human endothelial colony forming cells (ECFCs; Lonza, Basel, Switzerland) were grown in EGM-2 Microvascular Endothelial Cell Growth Medium (Lonza) with addition of an extra 5% FBS for ECFCs. ECFCs were grown in type I collagen-coated flasks (Thermo Scientific, Rockford, IL); all other cells were grown in uncoated tissue-culture treated flasks. Cells were maintained in a humidified atmosphere at 37°C and 5% CO_2_.

### Transduction with shRNA constructs

For shRNA knockdown prior to *in vitro* or *in vivo* analysis, U-87 MG or ECFCs were transduced (MOI 9) with individual clones or equivalent amounts of five pooled clones of MISSION shRNA lentiviral transduction particles (non-target control: SCH202V; SP1: NM_138473: TRCN0000020444, TRCN0000020445, TRCN0000020446, TRCN0000020447, TRCN0000020448; VEGFR2: NM_002253: TRCN0000195236, TRCN0000196398, TRCN0000001686, TRCN0000001687, TRCN0000001688; FGFR1: NM_000604: TRCN0000000418, TRCN0000121102, TRCN0000121106, TRCN0000121185, TRCN0000121307; FOXO1: NM_002015: TRCN0000010333, TRCN0000039579, TRCN0000039580, TRCN0000039581, TRCN0000039582, all from Sigma-Aldrich, St. Louis, MO) in growth media containing 8 µg/ml protamine sulfate (Sigma-Aldrich) for 72 h prior to selection with 5 µg/ml puromycin and screening for protein knockdown by Western blot analysis as described below. For shRNA knockdown concurrent with cord formation assay, ADSC/ECFC co-cultures were transduced 4 h following ECFC plating in cord formation in co-culture media (described below) containing 8 µg/ml protamine sulfate (Sigma-Aldrich) with 30 µl individual clones or equivalent amounts of five pooled MISSION shRNA lentiviral transduction particle clones (clone ID listed above) for 72 hours prior to analysis for Western blot or cord formation (described below). For determination of transduction efficiency, all cell types were transduced using a lentiviral-driven GFP construct (Sigma; Mission TurboGFP, # SHC003V). Seventy two hours post infection cells were placed under selective pressure using 2 µg/ml puromycin and GFP was measured using the Cellomics ArrayScan VTI, using the Target Activation bioapplication.

### Western blot analysis

Whole-cell protein extracts were isolated by cell lysis with 1% sodium dodecyl sulfate (SDS), and brief sonication. Protein concentration was quantified using the Bradford method. To separate proteins, 25 µg of whole-cell lysate was subjected to electrophoresis on 4 to 20% pre-cast Tris-glycine gradient gels (Invitrogen, Grand Island, NY) and transferred to nitrocellulose membranes (Invitrogen). The membranes were probed with primary antiserum, washed with Tris-buffered saline containing 0.1% tween (TBST), and incubated with an appropriate horseradish peroxidase-labelled secondary antibody. Membranes were washed with TBST, and signal was detected using an ECL kit (Thermo Fisher Scientific, Fremont, CA). Antibodies were diluted in TBST containing 5% blotting grade blocker (Biorad, Hercules, CA). The following antisera were used for immunoblotting: VEGFR2, FGFR1, FOXO1, SP1 (all from Cell Signaling Technologies, Beverly, MA), and β-actin (Santa-Cruz Biotechnology, Santa Cruz, CA). Densitometry was performed using Image J analysis software (NIH) as per the request of the Image J developers.

### *In vitro* Cord Formation Assay

As consistency in cord formation is an absolute necessity in evaluating gene knockdown, it is imperative that low passage number cell banks for each of the components be assembled prior to experimentation. ECFCs (passage 4–10 suitable for cord formation; the signal degrades with each subsequent passage beyond passage 10) were passaged onto type I collagen (fibrillar) coated flasks prior to seeding into the cord formation assay *in vitro*. ADSCs (Zen-Bio, cells frozen at passage 4; cells at passage 5 or greater not tested in our system) were plated at 75,000 cells per well into 96-well HTS Transwell (Corning, Lowell, MA) receiver plates (tumor-driven) or 50,000 cells per well (growth factor-driven) into 96-well black poly-D-lysine coated plates, and tumor cells were plated at 25,000 cells per well in 96-well HTS Transwell (Corning) plates in co-culture media [MCDB-131 media (Invitrogen) supplemented with L-ascorbic acid 2-phosphate, dexamethasone, tobramycin, insulin (all from Sigma-Aldrich), and cell prime r-transferrin AF (Millipore, Billerica, MA)] for 24 h. ADSC media was removed and 6,000 (tumor-driven) or 5,000 (growth factor-driven) ECFCs (Lonza) per well were over seeded. Treatment with 10 ng/ml VEGF or 15 ng/ml bFGF occurred 4 h following ECFC plating or 72 h following transduction with shRNA and continued for 72 h. Cells were directly fixed for 10 min with 3.7% formaldehyde (Sigma Aldrich) followed by ice-cold 70% ethanol for 20 min at 25°C. Cells were rinsed once with PBS, blocked for 30 min with 1% BSA and immunostained for 1 h with antiserum directed against CD31 (R&D Systems, Minneapolis, MN) diluted to 1 µg/ml in 1% BSA. Cells were washed 3 times with PBS and incubated for 1 h with 5 µg/ml donkey α-sheep-Alexa-488 (Invitrogen), α-Smooth Muscle Actin Cy3 conjugate (1∶200, Sigma-Aldrich), and 200 ng/ml Hoechst 33342 (Invitrogen) in 1% BSA, washed with PBS, then imaged using the cord formation and nuclei counts (for viability) algorithms on the Cellomics ArrayScan VTI at an image magnification of 5× (Thermo Fisher Scientific, Pittsburgh, PA). The tumor-driven cord formation assay, which is a modification of the growth factor co-culture assay, was performed as described previously [39]. Briefly, 2.5×10^4^ tumor cells were plated in the upper chamber of a HTS Transwell 96-well plate (Corning) that contains a 0.4 micron filter and the ADSC/ECFC were co-cultured in the bottom receiver chambers. After 72 hours, the receiver plates were fixed with 4% paraformaldehyde and stained as described for the growth factor driven assay.

For assessment of U-87 MG proliferation, HTS Transwell plates (Corning) were removed prior to receiver plate imaging and analyzed by Cell Titer Glo Luminescent Cell Viability Assay (Promega, Madison, WI) according to the manufacturer's recommendations.

### Cytokine Analysis

U-87 MG cells (2×10^5^) were plated in co-culture media in 6-well tissue culture dishes for 72 h prior to media collection and cell number counts. Cell debris was removed from conditioned media by centrifugation, and samples were analyzed fresh or were frozen at -20°C until analysis. Samples were analyzed with VEGF Quantikine Colorimetric Sandwich ELISAs (R&D Systems, Minneapolis, MN) according to the manufacturer's recommendations.

### *In vivo* Matrigel Plug Angiogenesis Assay

*In vivo* studies were carried out under Lilly Institutional Animal Care and Use Committee (IACUC) approved protocols. ADSCs (0.5×10^6^) were mixed with either non-transduced (sunitinib subset) ECFCs (2×10^6^) or ECFCs (2×10^6^) transduced with non-target control, VEGFR2, or FOXO1 shRNA (described above) and mixed with 200 µl growth factor reduced Matrigel (Becton, Dickenson and Company, Bedford, MA) on ice then subcutaneously injected into the flanks of athymic nude female mice (Harlan, Indianapolis, IN), two implants per animal. A subset of mice were dosed orally twice daily with sunitinib (25 mg/kg), which was prepared internally, beginning 4 hours prior to cell implantation. After 5 days of dosing, implants were excised, and placed in zinc-tris fixative (Becton, Dickenson and Company) for immunohistochemical analysis or flash frozen in liquid nitrogen, and hemoglobin was quantified using the QuantiChrom Hemoglobin Assay Kit (Bioassay, Hayward, CA) as previously described [20]. After 24 h in fixative, tumors were trimmed, embedded in paraffin blocks, and 4 micron sections were made. Slides were baked at 60°F for 1 hr and then deparaffinized in xylene (4×10 minutes); rehydrated with ethanol/water immersions with final washes in TBST; blocked with Protein Block (Dako, Carpinteria, CA) for 30 min; stained with a combination of Hoechst 33324, rabbit anti-GLUT1 (Dako)/anti-rabbit Alexa Fluor- 647 (Invitrogen), rat anti-human CD31 (Becton, Dickenson and Company)/anti-rat Alexa Fluor-488 (Invitrogen), and mouse anti-Smooth Muscle Actin/Cy3 (Sigma); imaged using an iCys Laser Scanning Cytometer (CompuCyte, Westwood, MA) and a Marianas Digital Imaging Workstation configured with a Zeiss Axiovert 200 M inverted fluorescence microscope (Intelligent Imaging Innovations, Denver, CO). Total vessel area was calculated as the percentage of total tissue area (Hoechst positive) that is also CD31 positive. Quantitative data comparisons of treatment groups were done using the Dunnett's analysis in JMP statistics software (SAS).

### *In vivo* pharmacology and tumor tissue immunofluorescence

U-87 MG (5×10^6^) cells either non-transduced (sunitinib subset) or transduced with non-target control or SP1 shRNA (Sigma-Aldrich; non-target: SCH202V; SP1: NM_138473) (described above) were mixed 1∶1 with Matrigel (Becton, Dickenson and Company, Bedford, MA) on ice and 200 µl was subcutaneously injected into the flanks of athymic nude female mice (Harlan, Indianapolis, IN), one implant per animal. A subset of mice was dosed orally once daily with sunitinib (40 mg/kg), which was prepared internally, beginning when tumors reached 300 mm^3^. After 6 days of dosing, tumors were excised and part was placed in zinc-tris fixative (Becton, Dickenson and Company) for immunohistochemical analysis (described above) while part was flash frozen in liquid nitrogen, and hemoglobin was quantified as described above.

### Statistical analysis

Statistical significance was assessed by a two-tailed Student *t* test with equal variance compared to the data for non-targeting shRNA control vector. Statistical significance was assigned to *p* values <0.05.
